# Clinical analysis of special types of tracheobronchial foreign bodies in children

**DOI:** 10.3389/fped.2024.1395629

**Published:** 2024-07-17

**Authors:** Hao Cai, Jinjian Gao

**Affiliations:** Department of Otorhinolaryngology, The Second Affiliated Hospital and Yuying Children’s Hospital of Wenzhou Medical University, Wenzhou, Zhejiang, China

**Keywords:** tracheobronchial foreign body, special types of foreign bodies, children, diagnosis, treatment

## Abstract

**Objectives:**

To explore the clinical diagnosis and treatment of special types of tracheobronchial foreign bodies in children and provide a reference for clinicians to formulate treatment plans.

**Methods:**

Clinical data of 29 children with special types of tracheobronchial foreign bodies who were treated at The Second Affiliated Hospital and Yuying Children's Hospital of Wenzhou Medical University between June 2017 and June 2022 were collected and analyzed, and their diagnosis and treatment processes were reviewed.

**Results:**

All 29 special types of foreign bodies were successfully removed using rigid bronchoscopy under general anesthesia, with no surgical complications.

**Conclusions and significance:**

For the treatment of special types of tracheobronchial foreign bodies, clinicians should make detailed surgical plans and select appropriate instruments according to different conditions to improve the surgical success rate and reduce the occurrence of complications.

## Introduction

Tracheobronchial foreign bodies are common and critical conditions in pediatric otolaryngology. If not treated promptly and accurately, it can lead to serious complications and even death. Tracheobronchial foreign bodies occurs primarily in children 1–3 years of age (1, 2). The chewing functions of infants and young children are imperfect, causing difficulties in chewing items. Moreover, children in this age group prefer to place small items into their mouths to play, which can easily lead to aspirations. The most common types of foreign objects are plant-based objects such as peanuts, melon seeds, and beans (3). In addition, there are some special types of foreign objects, such as plastic pen caps, glass balls, toys, metal foreign objects, and baby teeth. Owing to the unique shape and location of these foreign bodies, the difficulty of removal surgery has increased. For special types of tracheobronchial foreign bodies, mastering the key points of diagnosis and treatment and preparing for emergency situations during the perioperative period are extremely important for reducing complications and mortality rates. Here, we aimed to analyze and summarize 29 cases of special types of tracheobronchial foreign bodies in children admitted to our department in recent years.

## Patients and methods

### Patient information

The clinical data of 29 children with special types of tracheobronchial foreign bodies who underwent surgical treatment at The Second Affiliated Hospital and Yuying Children's Hospital of Wenzhou Medical University between June 2017 and June 2022 were included in the study. A detailed medical history was obtained preoperatively, and a history of foreign body coughing was confirmed in 29 patients. All patients underwent preoperative chest computed tomography (CT) examination before surgery.

### Methods

**Figure 1:**
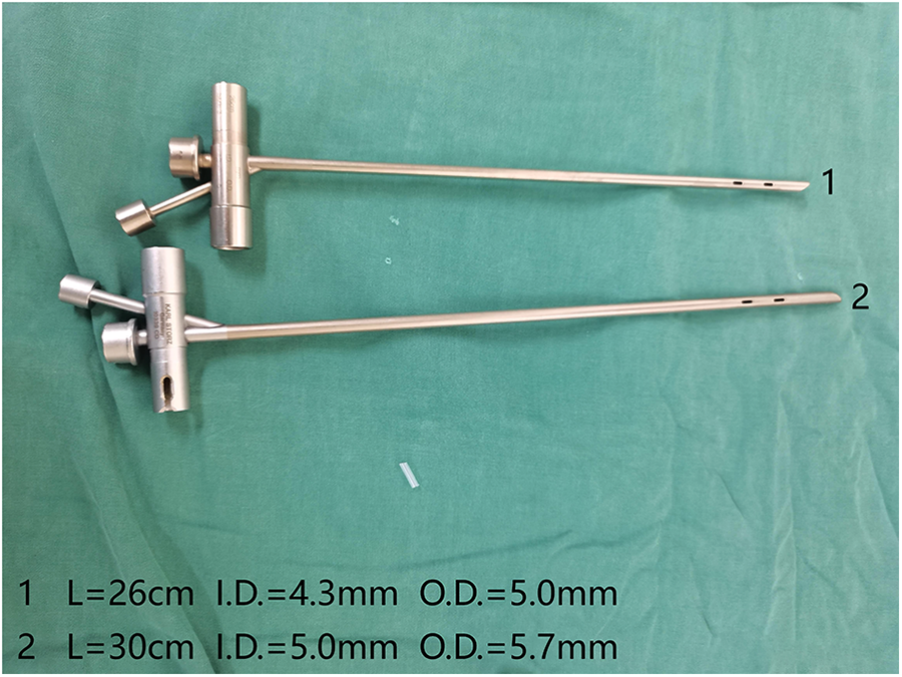
Rigid bronchoscope.

**Figure 2:**
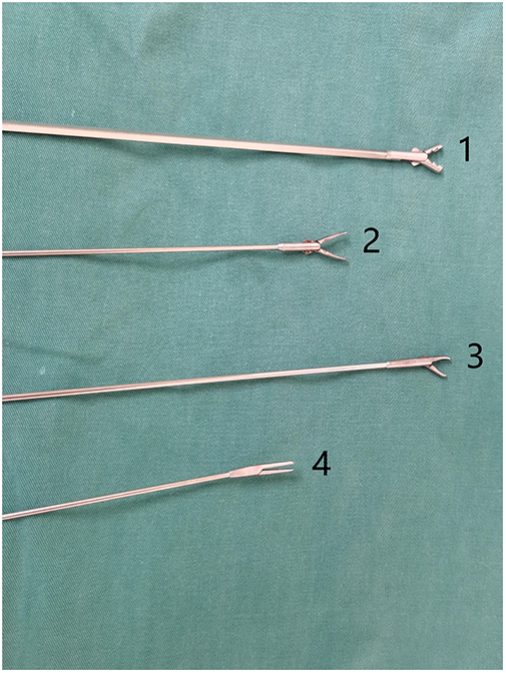
Foreign body forceps.

After general anesthesia, the patient was placed in a supine position with the head extended backward as much as possible. The patient was prepared and draped in the usual sterile fashion. First, a supportive laryngoscope was used to lift and fully expose the epiglottis. Tetracaine (1%) was applied to the surface of the glottis for anesthesia, and a rigid bronchoscope was placed (Figure 1). High-frequency-assisted ventilation was performed through the side hole of the bronchoscope, and the foreign body was observed. Suitable foreign body forceps were then selected to quickly remove the foreign body, clean the secretions in the trachea, and explore the healthy side of the trachea to ensure that no foreign body remained. We used foreign body forceps during the surgery as shown in Figure 2. We choose different forceps based on the actual situation during the surgical process. Conventional special types of foreign bodies usually choose forceps No.2 and No.4. Sharp or hard foreign bodies usually choose forceps No.1 and No.3. During this period, the vital signs of the child were closely monitored.

## Results

**Figure 3:**
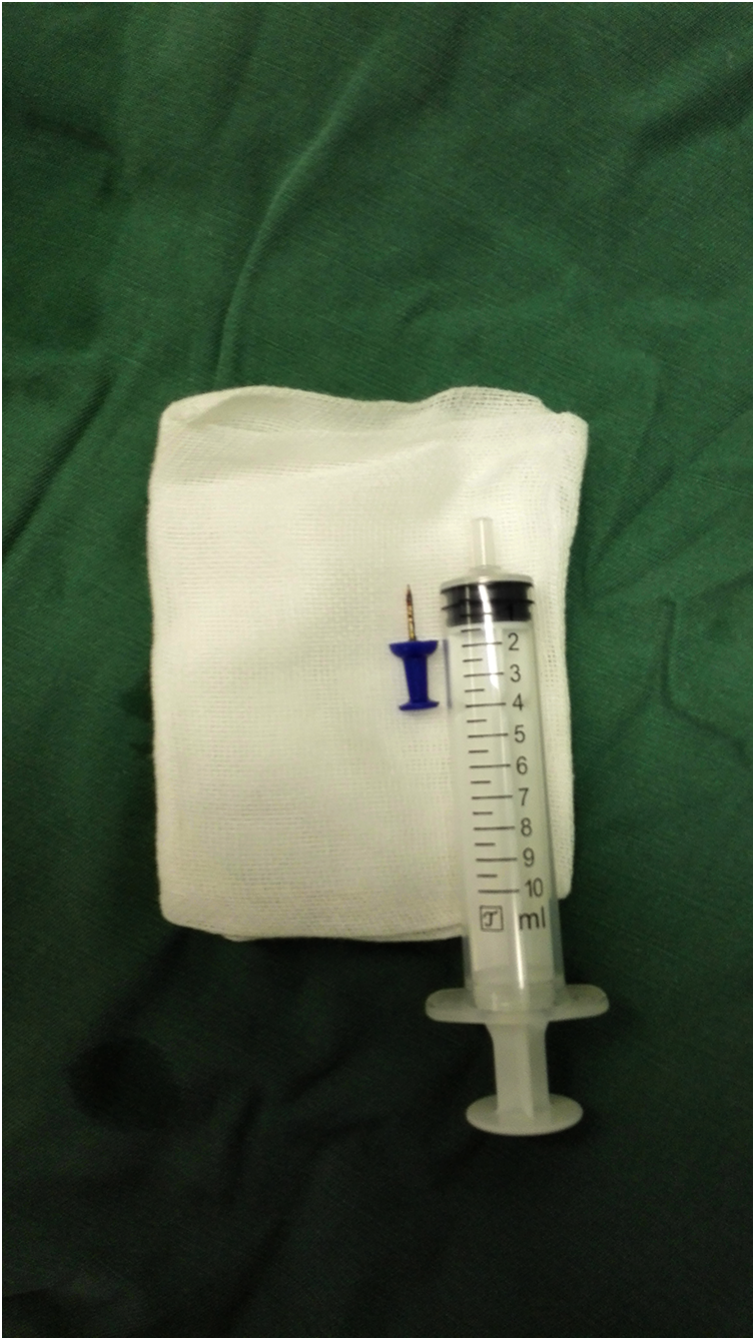
Thumbtack.

**Figure 4:**
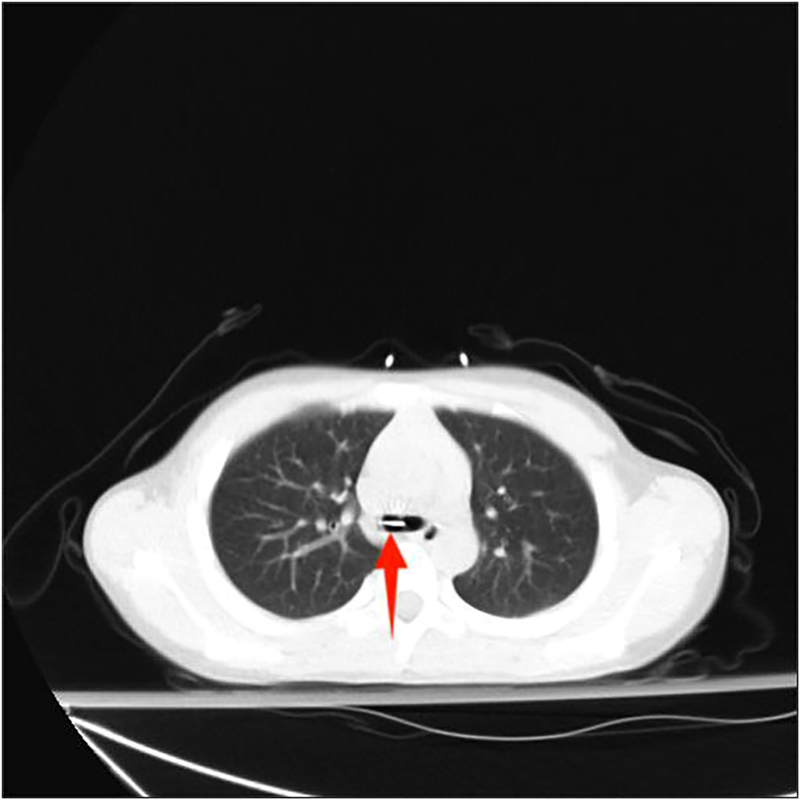
Thumbtack.

**Figure 5:**
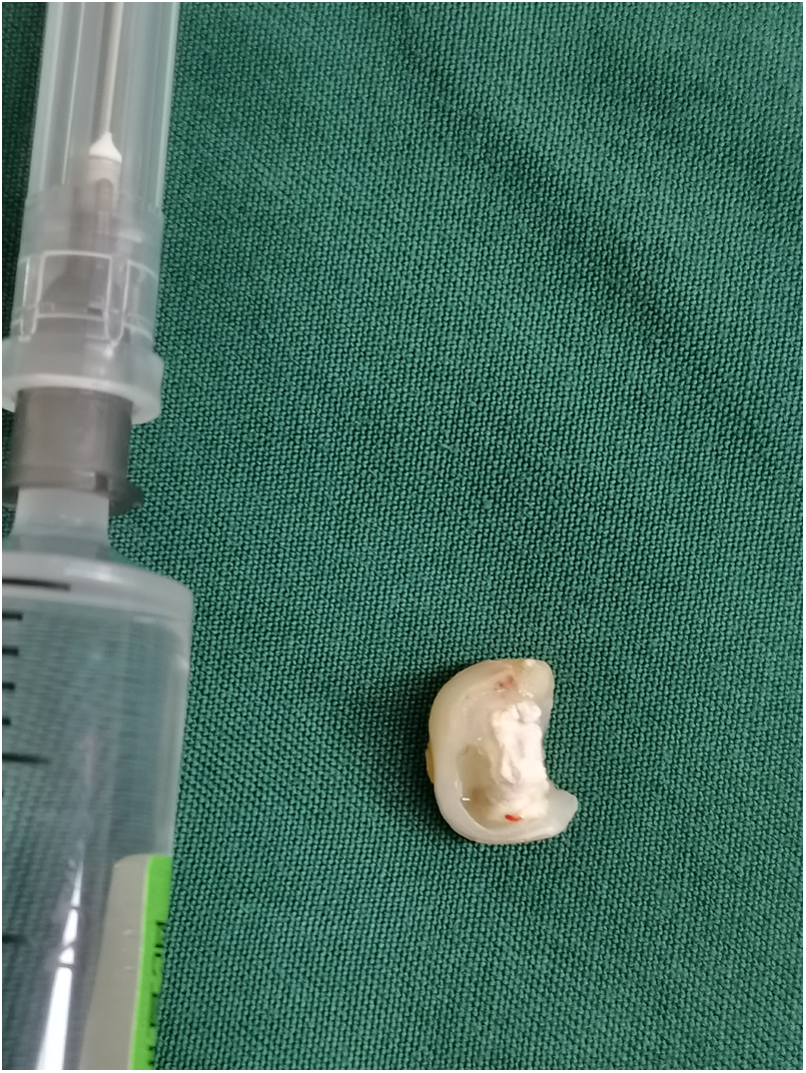
Tooth.

**Figure 6:**
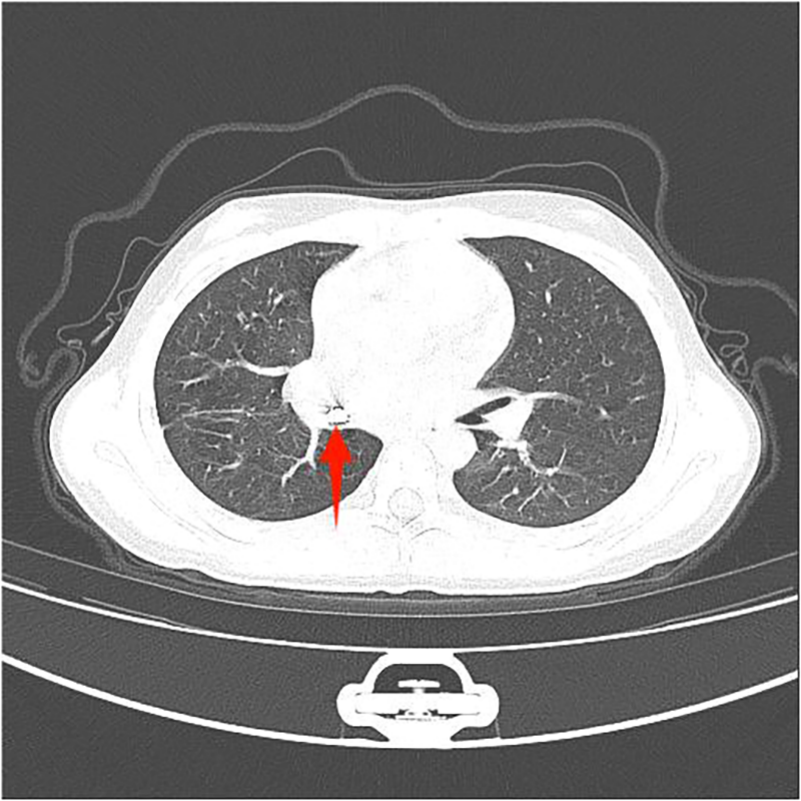
Tooth.

**Figure 7:**
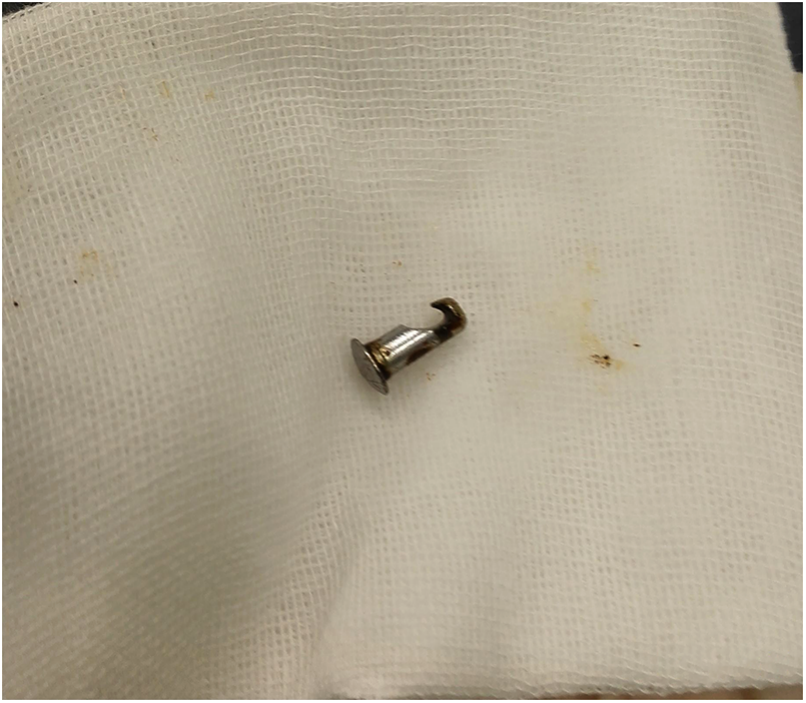
Metal foreign body.

**Figure 8:**
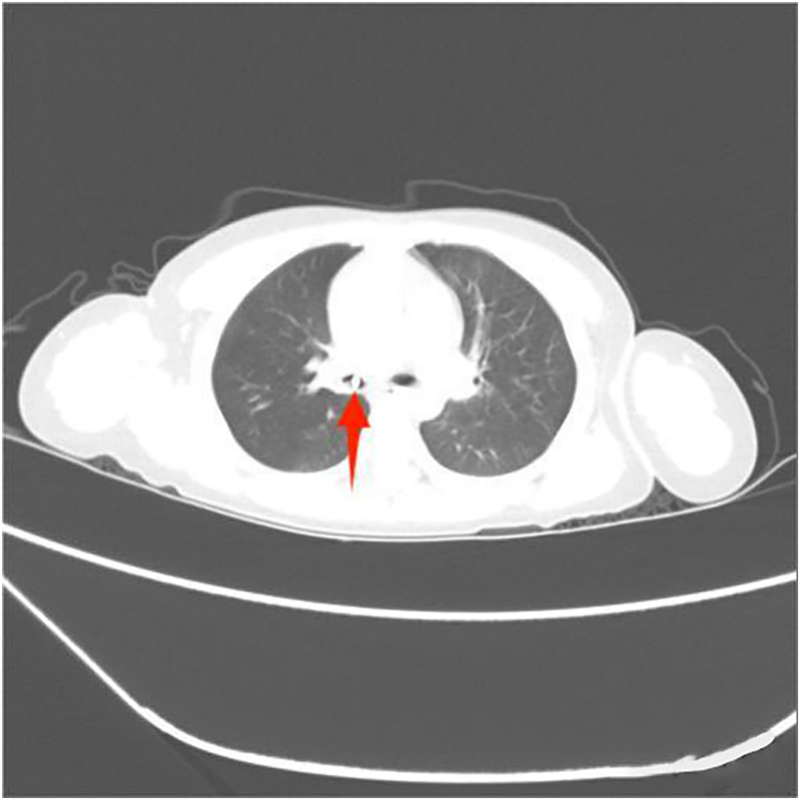
Metal foreign body.

Among the 29 patients included, the age ranged 8 months to 11 years, and 21 were males. The longest duration of foreign object choking was over 40 days, and the shortest was 1 h. There were 17 cases of foreign bodies in the right bronchus, 9 cases in the left bronchus, and 3 cases in the main trachea. The foreign bodies included eight plastic pen caps, four plastic fragments, four toys, two whistles, two thumbtacks, two metal foreign bodies, two stones, one mechanical spring, one diode, one tooth, one root canal treatment needle, and one eraser. Some foreign bodies are shown in the following image (Figures 3–8).

Bronchoscopy in all 29 patients was completed successfully without any surgical complications. After foreign body extraction, no patients were admitted to the ICU, and none died. After bronchoscopy, symptomatic supportive treatments, such as anti-infection, phlegm reduction, and nebulization, were administered. After the pulmonary symptoms improved, the patient was discharged from the hospital.

## Discussion

In the United States, the incidence of respiratory foreign bodies is 6.6/100,000 children (4). Medical history is most important for diagnosing tracheobronchial foreign bodies in children and usually manifests as sudden suffocation or severe coughing during eating or playing. Physical examination of a patient with a bronchial foreign body will reveal a decrease in respiratory sounds on one side, whereas when the foreign body is placed in the trachea, examinations will demonstrate equal breathing sounds bilaterally, with an impact sound heard in the upper chest in front of the trachea during coughing. Shortening the prehospital time is key to the diagnosis and treatment of tracheobronchial foreign bodies, as prolonging the prehospital time increases the risk of complications (5). Foreign bodies entering the lower respiratory tract can often cause pneumonia and bronchitis and are often treated as pneumonia in pediatric respiratory departments with anti-infection treatment (6). The main reason for misdiagnosis is the lack of a clear history of foreign body inhalation, which may be related to an incomplete medical history provided by the patient's family members or insufficient detailed collection of medical history by the clinicians. Early diagnosis and treatment are extremely important for reducing the incidence and mortality in children (7, 8). If there is a clear history of foreign body aspiration, the possibility of a tracheal foreign body should be considered when the symptoms, signs, and imaging findings are atypical. Compared with chest radiography, three-dimensional chest CT and CT reconstruction have higher sensitivity, and the missed diagnosis rate can be significantly reduced (9). When a patient's vital signs are stable, CT and CT three-dimensional reconstruction should be the first choice to reduce the probability of misdiagnosis (10, 11). Simultaneously, CT can provide information on the location, size, shape, nature, and surrounding inflammation of foreign bodies, making it easier to evaluate the condition and plan the procedure.

The treatment principle for tracheobronchial foreign bodies is surgical removal as early as possible. Rigid bronchoscopy under general anesthesia is the primary surgical method for removing special types of respiratory foreign bodies (12). For routine tracheobronchial foreign bodies, especially plant-based foreign bodies, fexible bronchoscopy is also an effective and convenient surgical method. But in cases of clinical instability in patients, or when large or sharp special types of foreign bodies are found, rigid bronchoscopy is usually more suitable and safe (13). Preoperative patients should strictly abstain from food and water and avoid exercise and intense crying to prevent changes in the position of foreign bodies. The timing of bronchoscopy depends on the severity of the patient's condition upon admission, duration of foreign body aspiration, and presence of complications. If breathing difficulties occur, emergency surgical treatment is required; if there is no obvious difficulty in breathing, it is best to make sufficient preoperative preparations.

Special types of tracheobronchial foreign bodies, owing to their unique shape and large volume, generally require general anesthesia. General anesthesia can reduce restlessness in children, relax the smooth muscles in the trachea, and facilitate the removal of foreign bodies. However, general anesthesia should reach enough depth to achieve muscle relaxation, fewer secretions, and pain relief when placing a rigid bronchoscope and removing a foreign body through the glottis (14). The success of removal is closely related to the cooperation of the anesthesiologists (15). The procedure should be performed by experienced surgeons who communicate well with the anesthesiologists to cope with the various situations that may arise during the surgical process.

The key to removing tracheobronchial foreign bodies lies in whether the surgical method is appropriate and whether the surgeon has enough experience. Before bronchoscopy, it is necessary to fully understand the size, nature, and shape of the foreign bodies; prepare corresponding instruments for different types of foreign bodies; and design targeted removal methods. Sufficient pre-operative preparation and emergency rescue measures during the procedure are necessary to ensure patient safety. For special types of tracheobronchial foreign bodies, obtaining physical objects before bronchoscopy and performing simulated operations *in vitro* can significantly reduce intraoperative risk. If the nature of the foreign body is unclear or cannot be obtained before bronchoscopy, the secretions should be thoroughly cleared before clamping the foreign object, and the shape of the foreign object and its relationship with the surrounding trachea should be carefully observed. A suitable foreign-object clamp should be selected to clamp the appropriate part of the foreign object and should not be pulled blindly or forcefully. This avoids damaging the tracheal mucosa, prevents losing grip on the foreign object, and reduces complications.

For larger foreign bodies, such as pen caps and erasers, the gap between the tracheal wall and the foreign object should be determined. During bronchoscopy, if there is no gap between the tracheal wall and foreign object, adrenaline-soaked cotton pads should be used to constrict the tracheal mucosa. After finding the gap, the foreign body should be grasped and gently shaken to allow the gas to enter the distal bronchus and reduce the negative pressure. When passing through the glottis, the bronchoscope lip should be faced downward and pressed down the anterior joint to expand the glottis and allow the foreign object to exit the throat as far as possible from the glottic fissure. When the volume of the foreign body is too large to pass through the glottis, a tracheostomy should be performed to remove the foreign body rather than simply clamping it, which can cause serious iatrogenic trauma to the child and even surgical failure (16).

When removing sharp foreign bodies, such as thumbtacks and root canal treatment needles, the sharp tip should be faced toward the inside of the bronchoscope or toward the distal end, making the needle body aligned with the longitudinal axis of the bronchoscope. In particular, scratching of the tracheal mucosa should be avoided during removal as it may cause complications including bleeding, pneumothorax, and mediastinal emphysema. Local trauma caused by an irregular and sharp foreign body, and extended period of time represent the main factors causing granuloma formation (17). In this study, two patients were found to have granulomas during surgery. We removed them and used adrenaline cotton balls to compress and stop bleeding.

Hollow foreign bodies, such as whistles, usually do not cause complications such as atelectasis; however, these types of foreign bodies often adhere tightly to the walls of the trachea and bronchi. After locating the gap, the foreign body should be tightly grasped with forceps and removed using a bronchoscope. Reverse-tensioning pliers can be used for an easier and safer removal. Caution should be exercised when passing through the glottis. If resistance is present, the foreign body clamp can be slowly rotated to allow the foreign body to exit the throat at the optimal position of the glottis.

If a large, irregular foreign body accidentally slips off when passing through the glottis, it can suddenly cause suffocation, cyanosis, and a sharp decrease in blood oxygen pressure. Because the bronchus of the affected side has been blocked for a long time, the secretions remained, while there is significant negative pressure and suction in the bronchus of the healthy side. In such cases, the foreign body can fall off and occlude the healthy side, namely the “foreign body displacement.”If the healthy side is blocked by a foreign object, and gas cannot be exchanged, respiratory failure may occur immediately. At this point, the bronchoscope should be immediately inserted directly into the opposite side to search for foreign objects, which should be grasped, removed, or pushed to the distal end of the bronchus to alleviate breathing difficulties.

## Conclusion

In conclusion, special types of tracheobronchial foreign bodies are among the most critical emergencies in pediatric otolaryngology. Long-term clinical practice has proven the practical value of these methods. Due to the diversity of foreign bodies, no surgical instruments or methods can cover everything. Attention should be paid to the medical history before bronchoscopy, and a rapid evaluation should be made based on the nature of the foreign body and the general situation of the patient. A detailed surgical plan should be formulated and appropriate instruments should be selected to improve the success rate of the procedure and reduce the occurrence of complications.

## Data Availability

The raw data supporting the conclusions of this article will be made available by the authors, without undue reservation.

## References

[1] Samra S Schroeder JW Valika T Billings KR. Tracheotomy for difficult airway foreign bodies in children. Otolaryngol Head Neck Surg. (2018) 158(6):1148–49. 10.13201/j.issn.2096-7993.2024.02.014 29437526

[2] Na'ara S Vainer I Amit M Gordin A. Foreign body aspiration in infants and older children: a comparative study. Ear Nose Throat J. (2020) 99(1):47–51. 10.1177/0145561319839900 30974996

[3] Gang W Zhengxia P Hongbo L Yonggang L Jiangtao D Shengde W Diagnosis and treatment of tracheobronchial foreign bodies in 1024 children. J Pediatr Surg. (2012) 47(11):2004–10. 10.1016/j.jpedsurg.2012.07.036 23163990

[4] Cheng J Liu B Farjat AE Routh J. The public health resource utilization impact of airway foreign bodies in children. Int J Pediatr Otorhinolaryngol. (2017) 96:68–71. 10.1016/j.ijporl.2017.03.009 28390617

[5] Gan W Xiao N Feng Y Zhou D Hu J Liu S Clinical analysis of tracheobronchial foreign body aspiration in children: a focus on external and intrinsic factors. BMC Surg. (2021) 21(1):108. 10.1186/s12893-021-01089-3 33658017 PMC7927229

[6] Zhijun C Fugao Z Niankai Z Jingjing C. Therapeutic experience from 1428 patients with pediatric tracheobronchial foreign body. J Pediatr Surg. (2008) 43(4):718–21. 10.1016/j.jpedsurg.2007.10.010 18405721

[7] Oncel M Sunam GS Ceran S. Tracheobronchial aspiration of foreign bodies and rigid bronchoscopy in children. Pediatr Int. (2012) 54(4):532–5. 10.1111/j.1442-200X.2012.03610.x 22414345

[8] Wang Y Sun Y Zhang H Yang X Song X. Comprehensive analysis of the diagnosis and treatment of tracheobronchial foreign bodies in children. Ear Nose Throat J. (2023) 102(10):661–6. 10.1177/01455613211023019 34112007

[9] Huang HJ Fang HY Chen HC Wu CY Cheng CY Chang CL. Three-dimensional computed tomography for detection of tracheobronchial foreign body aspiration in children. Pediatr Surg Int. (2008) 24(2):157–60. 10.1007/s00383-007-2088-2 18040695

[10] Shin SM Kim WS Cheon JE Jung AY Youn BJ Kim IO CT in children with suspected residual foreign body in airway after bronchoscopy. AJR Am J Roentgenol. (2009) 192(6):1744–51. 10.2214/AJR.07.3770 19457843

[11] Bai W Zhou X Gao X Shao C Califano JA Ha PK. Value of chest CT in the diagnosis and management of tracheobronchial foreign bodies. Pediatr Int. (2011) 53(4):515–8. 10.1111/j.1442-200X.2010.03299.x 21129123

[12] Cutrone C Pedruzzi B Tava G Emanuelli E Barion U Fischetto D The complimentary role of diagnostic and therapeutic endoscopy in foreign body aspiration in children. Int J Pediatr Otorhinolaryngol. (2011) 75(12):1481–5. 10.1016/j.ijporl.2011.08.014 21924505

[13] De Palma A Brascia D Fiorella A Quercia R Garofalo G Genualdo M Endoscopic removal of tracheobronchial foreign bodies: results on a series of 51 pediatric patients. Pediatr Surg Int. (2020) 36(8):941–51. 10.1007/s00383-020-04685-1 32468145

[14] Liu Y Chen L Li S. Controlled ventilation or spontaneous respiration in anesthesia for tracheobronchial foreign body removal: a meta-analysis. Paediatr Anaesth. (2014) 24(10):1023–30. 10.1111/pan.12469 24975102

[15] Loreau C Caruselli M Roncin C Salvi N Lenoire A Allary C Pediatric anesthetic for tracheobronchial foreign body extraction: a survey of practice in France. Paediatr Anaesth. (2023) 33(9):736–45. 10.1111/pan.14704 37300331

[16] Fraga JC Neto AM Seitz E Schopf L. Bronchoscopy and tracheotomy removal of bronchial foreign body. J Pediatr Surg. (2002) 37(8):1239–40. 10.1053/jpsu.2002.34493 12149717

[17] Zhenghua H Ai Z Jianya Z Lisheng X Qi L. Risk factors for granuloma formation in children induced by tracheobronchial foreign bodies. Int J Pediatr Otorhinolaryngol. (2015) 79(12):2394–7. 10.1016/j.ijporl.2015.10.057 26596356

